# Supplementary material for: Oral creatine in hemodialysis patients increases physical functional capacity and muscle mass, an open label study
Source: PLoS One. 2025 Jul 31;20(7):e0328757. doi: 10.1371/journal.pone.0328757 (PMC12312878; doi:10.1371/journal.pone.0328757)
Supplement: S1 Table — Mean difference (post – pre): 0.85 [95% CI: 0.00–1.54], based on 10,000 bootstrap replicates of the paired differences. (DOCX) [file pone.0328757.s001.docx]

**Supporting information**

**S1 Table: SPPB score pre and post creatine supplementation in patients with increased vitamin D levels.**

| SPPBpre | SPPBpost |
| --- | --- |
| 12 | 12 |
| 10 | 11 |
| 12 | 12 |
| 10 | 12 |
| 5 | 9 |
| 7 | 9 |
| 12 | 12 |
| 10 | 12 |
| 11 | 12 |
| 11 | 12 |
| 12 | 12 |
| 12 | 12 |
| 10 | 8 |
| 12 | 12 |

Mean difference (post – pre): 0.85 [95% CI: 0.00 – 1.54], based on 10,000 bootstrap replicates of the paired differences.
