# Supplementary material for: Oral creatine in hemodialysis patients increases physical functional capacity and muscle mass, an open label study
Source: PLoS One. 2025 Jul 31;20(7):e0328757. doi: 10.1371/journal.pone.0328757 (PMC12312878; doi:10.1371/journal.pone.0328757)
Supplement: Attachment — Submitted filename: Response to reviewers.Final.20jun25.docx [file pone.0328757.s005.docx]

**Rebuttal letter**

Coyhaique, June 20th, 2025

Dear editor and reviewers,

We would like to thank you for the thoughtful and constructive review of our manuscript. The observations and suggestions provided have certainly prompted us to improve and enrich our paper, making it more rigorous and scientifically robust. In the next paragraphs you will find our responses in the same order as we received the observations and answered point by point. We hope we have been able to respond clearly to each of your comments and suggestions.

MAJOR DETAILS:

1. The structure of the manuscript, especially the METHODS section, needs to be revised. All subsections should be organized in strict order as recommended by CONSORT guidelines for pilot and feasibility studies (Trial design, Participants, Intervention Procedures, Outcomes, Sample Size, Statistical Analysis) as informed in the submission guidelines of PLoS One. Cf, PMID 31608150 or [www.equator-network.org](http://www.equator-network.org).

**Answer 1:** We thank the editor for raising this point.

During the process of designing our study we consulted the NIH web page: Decision Tool: Does Your Human Subjects Study Meet the NIH Definition of a Clinical Trial? (<https://grants.nih.gov/policy-and-compliance/policy-topics/clinical-trials/ct-decision>). After careful consideration we thought that our study didn’t meet the “four questions to determine if your study is a clinical trial”, and for this reason we decided to report it as an observational study, following the STROBE guidelines. Although, we agree with the editor, that this study can be also considered an experimental study (a “single-arm repeated-measures feasibility clinical trial”). The revised version of our paper has changed the methods sections and now presents the subsections organized as recommended by CONSORT guidelines (please see CONSORT checklist).

Was the clinical trial registered? Platforms such as ClinicalTrials.gov, OSF, or Figshare are available for this purpose.

**Answer 2:** Since we did not consider our study to be a clinical trial, it was not enrolled in any of the respective platforms, before starting patient recruitment. We are currently in the process of enrolling our study *a posteriori*, on clinicaltrials.gov platform (Universidad de Aysén Protocol Record ORD252023, in process). We also reviewed the other two alternatives proposed by the editor (Figshare and OSF), however, these appear to be platforms for sharing data and projects during their development, but did not have the features shared by the platforms suggested by the World Health Organization, grouped in the International Clinical Trials Registry Platform (ICTRP).

As our institution was not previously enrolled in clinicaltrials.gov platform, the process for the institution to be registered and the posterior steps took more time that the one considered for answering the reviewers comments. For this reason, the clinicaltrials.gov enrollment process is still ongoing. We understand this information is important and still pending. As soon as the registration is complete, we will send the information.

Were participants aware that they were receiving the creatine monohydrate supplement (i.e., open-label study), or were they blinded to the intervention (i.e., single-blind)? Consider including this information in the title for greater clarity.

**Answer 3:** All participants were aware that they were receiving creatine. We included the phrase 'open-label' in the title to clarify this point, and also mentioned it in the creatine supplementation section of the Methods (lines 123-124, clean version).

This study is not a prospective observational study. This is a single-arm repeated-measures feasibility clinical trial (revise Line 126 and line 300). This is an experimental study.

**Answer 4:** We agree with the editor. Please see our more complete response to this issue, in the comment above (“answer 1”). We have made the changes accordingly.

The authors should clarify which variables are considered primary outcomes and which are secondary outcomes.

**Answer 5:** We thank the editor for highlighting this important point and allowing us to clarify these aspects.

The primary outcome of this study was to evaluate the physical performance of hemodialysis patients before and after receiving creatine supplementation as assessed by the Short Physical Performance Battery (SPPB).

The secondary outcomes in the current study were to demonstrate changes in muscle strength, measured by hand dynamometry, and changes in body composition, estimated by bioimpedance analysis (skeletal muscle mass, fat free mass, total body water, extracellular water and intracellular water). Additionally, we aimed to evaluate changes in phase angle before and after eight weeks of creatine supplementation.

This information has been explicitly included in the Materials and Methods section, (lines 125-135, clean version).

Was dietary intake or physical activity recorded during the study?

**Answer 6:** This is a very important point and we, deliberately, decided not to modify or register dietary intake nor physical activity**.** The former decision was taken based on bibliography that demonstrates that self-monitoring has significant impacts on the measured variables inducing changes on dietary intake and physical activity (Burke LE, Wang J, Sevick MA. Self-monitoring in weight loss: a systematic review of the literature. J Am Diet Assoc., 2011: 111(1):92-102. doi: 10.1016/j.jada.2010.10.008; Tang, HB., Jalil, N.I.A., Tan, CS. *et al.* Why more successful? An analysis of participants’ self-monitoring data in an online weight loss intervention. BMC Public Health, 2024: 24, 322. doi.org/10.1186/s12889-024-17848-9).

We also did not recommend any changes to the participants' usual diet; instead, we maintained the general dietary recommendations routinely provided by the dietitian at our dialysis unit, primarily focusing on foods low in phosphate, sodium, and potassium (lines 239-241, clean version).

Regarding physical activity, it was not recorded in this first pilot study. We assessed physical performance exclusively using the Short Physical Performance Battery (SPPB). However, we are currently seeking funding to conduct a new study—a randomized, double-blind, multicenter trial with a larger patient population—in which we will include physical activity assessment using the International Physical Activity Questionnaire (IPAQ).

Please provide additional details regarding the blood sampling procedures, the test protocols used to evaluate biomarkers, and the equipment or devices employed in the laboratory.

**Answer 7:** The national regulations related to the care of patients in chronic hemodialysis, consider the evaluation of specific routine laboratory tests. Some of these tests are performed monthly, while others are carried out quarterly or semiannually. As in many other countries, it is standard practice to collect monthly blood samples prior to dialysis during the second session of the week, which typically falls on Wednesday or Thursday, depending on the patient's schedule. Additionally, post-dialysis blood samples are taken at the end of the same session, primarily to calculate the dialysis dose using widely accepted and standardized formulas such as Kt/V.

The blood samples collected in the dialysis unit are subsequently transferred to the hospital laboratory, which is located in the same building. Biochemical analyses—including electrolytes, calcium, parathyroid hormone (PTH), and vitamin D—are performed using the ARCHITECT ci4100 analyzer (Abbott). Complete blood counts are analyzed using the CELL-DYN Ruby system (Abbott), (lines 149-150, clean version).

Have the formulas been validated in the study population, or at least in a Latin American population? If not, this should be acknowledged as a limitation.

**Answer 8:** We thank the editor for pointing this out, as we had mistakenly assumed it was a general topic when it is actually part of the specialized technical knowledge handled primarily by professionals working in dialysis units every day. The following is a brief description of the history and use of each of these parameters. For the sake of clarity in the text, we have incorporated a clarifying paragraph and included the article (lines 468 - 472, clean version).

The Kt/V and nPCR formulas are routinely used in dialysis units worldwide. These formulas have been extensively studied and analyzed since the 1980s, when efforts began to establish methods for quantifying dialysis dose and correlating it with patient mortality. Although, to our knowledge, they have not been specifically validated in Latin American populations, they are universally accepted without distinctions based on race or gender. The Kt/V formulas, such as those proposed by Daugirdas are based on objective, patient-specific parameters—including pre- and post-dialysis urea levels, dialysis duration, ultrafiltration volume, and post-dialysis body weight—and do not incorporate race or ethnicity as a variable. Similarly, the nPCR formula, which is used to assess protein catabolism in dialysis patients, also relies on objective measures, such as urea concentrations and body weight, and does not include any race-based adjustments. Unlike certain estimations of glomerular filtration rate (e.g., Modification of Diet in Renal Disease, MDRD), which historically included race-based adjustments, both Kt/V and nPCR are applied uniformly across populations. Therefore, the application of both Kt/V and nPCR in our study population is consistent with international clinical practice.

Based on the aforementioned, we do not believe that the data obtained using these formulas could introduce any bias in the population included in our study.

2. Replace conventional bioelectrical impedance analysis with bioelectrical impedance vector analysis (BIVA). If you used the mBC 565 device, you should present BIVA results—such as the Xc versus R plot normalized by stature, or even specific BIVA using available body girth measurements—instead of relying solely on body composition estimates of SMM, ICW, FFM, ECW, and TBW. While phase angle is a valuable absolute outcome of BIVA, it can be further complemented with indices like the BI index (Height²/Z at 50 kHz) and the impedance ratio (Z at high and low frequencies in kHz).

"An IR ratio closer to 1 is indicative of cell membrane disruption, allowing more fluids, proteins, and electrolytes to shift into the extracellular space [22]. A strong inverse correlation has been reported between the phase angle (PhA) and IR in different clinical populations [22]. PhA is defined as the delay in current flow caused by a reduction in cell membrane capacitance [25]." Cf, PMID: 38488531

**Answer 9:** Thank you for your valuable comment regarding the inclusion of additional bioimpedance-derived parameters.

Regarding this point, and taking into account new information we obtained while addressing another of your suggestions—specifically the request to provide the coefficient of variation (CV%) of the bioimpedance device used—we have decided to modify the focus of our discussion.

Our search revealed that the only reported coefficient of variation (CV%) for the device refers to skeletal muscle mass, with values of 0.4% for intra-operator and 0.6% for inter-operator assessments. For estimated variables such as total body water (TBW) and extracellular water (ECW), studies in South American populations report a standard error of estimate (SEE) of 1.4 liters for TBW and 0.7 liters for ECW. (seca GmbH & Co. KG. *Instructions for use: seca mBCA 525 / seca mBCA 535*. Hamburg (Germany): seca GmbH; 2022. p. 126. Available from:<https://www.seca.com/>).

Therefore, and following your suggestion, we considered it was more appropriate to focus our analysis on variables that are either directly measured or supported by stronger evidence and smaller coefficients of variation. We also considered it important that the information derived from this and future studies be as clear and interpretable as possible for the multidisciplinary teams involved in dialysis care—as well as for the patients themselves. Accordingly, we are now proposing to center our analysis primarily on a variable that is directly calculated by the bioimpedance device—without requiring additional software or complex formulae—thus facilitating both data acquisition and clinical interpretation. This variable is **phase angle**, which is derived from raw measurements (resistance and reactance) and is provided directly in the device’s standard report, as is also the case with other commercial bioimpedance analyzers, without the need for further manual data processing.

As a second approach, we propose focusing on **skeletal muscle mass**, as its estimation via bioimpedance is supported by validated cut-off values and has been explored in prior studies—particularly in the context of creatine supplementation in dialysis patients (Marini, et al, see reference number 53). As a secondary analysis, we will describe the changes observed in body water compartments (TBW, ECW, and intracellular water [ICW]), acknowledging that these are less precise estimations.

Based on your suggestions we conducted a re-analysis, exploring additional indices such as the **bioimpedance index** or **impedance ratio.** Although valuable as a more direct representation of bioimpedance measurements, we found it difficult for those concepts to have a generalized clinical applicability. These metrics introduce concepts that may be difficult for healthcare teams and patients to interpret and, additionally, require a more exhaustive data extraction and processing effort by the clinical team.

Nevertheless, we conducted bootstrap analyses of the differences in resistance and reactance, and also calculated changes in the bioimpedance index using the same statistical approach. These results are attached in the following table, should you consider them appropriate for inclusion in the final manuscript version.

|  | Pre | post | Bootstrap mean difference [95% CI] |
| --- | --- | --- | --- |
| Bioimpedance Index | 860.37 | 900.80 | 40.43 [7.27 a 76.56] |
| Resistance/height | 295.76 | 282.37 | -13.4 [-25.64 a -2.26 ] |
| Reactance/height | 27.74 | 29.04 | 1.29 [-0.64 a 3.16 ] |

1 Point estimate and 95% confidence interval based on 10 000 bootstrap replicates of the pre–post differences.

2 Effect size estimates with 95% confidence intervals based on 10,000 bootstrap resamples

*Statistically significant at 5% level

3. Statistical analysis.
This section requires revision. Due to the small sample size and the fact that the required statistical power was not achieved (i.e., fewer participants than indicated by the sample size calculation), the authors should avoid null hypothesis significance testing (NHST). Instead, the analysis should focus on estimation methods—reporting 95% confidence intervals and unbiased effect sizes (e.g., Cohen's d, also known as Hedges' g)—as well as robust statistics, such as trimmed means and Winsorized standard deviations. Please re-run the analysis accordingly and confirm whether the findings and conclusions remain consistent. Thanks to your open data sharing, I have attached the raw data Excel file along with an example of the results output from Jamovi, which you may use for organizing and presenting your findings.

Cf,
- Estimation statistics:<https://pubmed.ncbi.nlm.nih.gov/24220629/>
"ESCI" module in Jamovi
- Robust statistics:<https://pubmed.ncbi.nlm.nih.gov/31152384/>
"Walrus" module in Jamovi
In addition, please generate and replace current figures with estimation plots to display the repeated measures data across time points (at baseline and after creatine supplementation). Cumming or Gardner-Altman estimation plots are recommended (see examples of these figures in the attached Excel file).

Please add the CV% of the instruments/technician to know the variability of all measures.

**Answer 10:** Thank you for your suggestions on this point, as they prompted us to delve into a subject we were previously unfamiliar with, robust statistics.

We agree that null hypothesis significance testing is a suboptimal approach for our case. Accordingly, we have removed all p-values. Due to the small sample size, trimming-based tests such as Yuen’s paired t-test resulted in a substantial reduction of effective data, leaving us with only 12 pairs of data to analyze.

Instead, given our small sample size, we are proposing to analyze our data by using another robust statistics approach, which uses bootstrap resampling (10 000 replications) to estimate pre-post differences. We chose this approach because it allows us to preserve all observations and avoid strong distributional assumptions. This is complemented by effect size estimation, which provides robust and clinically meaningful insights without reducing the sample size. We used Hedges' *g* to calculate effect size, as it is more appropriate for small samples (n < 20; see reference 23). We hope this novel approach to analysis will be deemed appropriate.

Our search revealed that the only reported coefficient of variation (CV%) for the device refers to skeletal muscle mass, with values of 0.4% for intra-operator and 0.6% for inter-operator assessments. (Bosy-Westphal, A., Jensen, B., Braun, W., Pourhassan, M., Gallagher, D., & Müller, M. J. (2017). Quantification of whole-body and segmental skeletal muscle mass using phase-sensitive 8-electrode medical bioelectrical impedance devices. *European journal of clinical nutrition*, *71*(9), 1061–1067).

MINOR DETAILS:

- Please adjust the background and hypothesis in the introduction section (as well as in the abstract) to creatine related information. The introduction can be reduced to one page only. Missing key references on this topic:

<https://pubmed.ncbi.nlm.nih.gov/31083291/>
<https://pubmed.ncbi.nlm.nih.gov/28110688/>
<https://pubmed.ncbi.nlm.nih.gov/34444869/>

**Answer 11:** We have implemented the suggested revisions in both the introduction and the abstract sections. We are grateful for the recommended references; one was already included in the original version of the article and we have now incorporated the remaining two (lines 92 - 95, clean version). See references 11,12 and 13.

- Although "body weight" and "height" are frequently used terms, it is technically correct to refer to "body mass" and "stature", respectively. Please address this accordingly throughout the manuscript.

**Answer 12**: Thank you. We have now completed the corrections.

- Dynamometer strength measurements should be expressed in kilogram-force (kgF). Please revise accordingly and replace "Strength" with "Handgrip Strength" in Figure 1.

**Answer 13**: Corrected

- Do not use "mean±standard deviation." Use Mean(SD) instead. Cf, PMID 21206631

**Answer 14:** In the tables, the results are expressed as mean (SD), as you suggested.

- The **results** of all variables should be expressed as Δ ± SD [95% CI of the change]; unbiased Cohen's [with the corresponding 95% CI]. Remember that unbiased Cohen's = Hedge's g

**Answer 15:** We revised the results to be presented in the manner suggested in the text.

- Please edit the tables to present all relevant findings, including effect sizes, 95% confidence intervals, and other pertinent statistics.

**Answer 16:** We added the columns to display all the relevant results you mentioned.

- Line 374: The authors should highlight that this is one of the first clinical trials to evaluate the effects of oral creatine monohydrate supplementation on BIVA variables, strength-related outcomes, and functional capacity in hemodialysis patients.

**Answer 15:** Thank you for your suggestions; we have revised the text as requested. See lines 487-489, clean version

- The discussion should place greater emphasis on BIVA-related data (e.g., bioimpedance indices reflecting cellular integrity), strength-related outcomes, and estimates of body composition (e.g., skeletal muscle mass, fat-free mass, etc.).

**Answer 16:** As pointed out in answer 9, we agree with the editor´s suggestion. Thus, in the updated text corresponding to the “discussion” section, these suggestions have been incorporated see lines 362- 463, clean version

- The discussion on intra- and extra-celullar water might be enriched if the impedance ratio is reported (see comments above).

**Answer 17:** We agree with this suggestion but, as the variables such as total body water (TBW) and extracellular water (ECW) were found to have a high SEE (answer 9) we finally decided not to include these results in the discussion section.

- In agreement with the reviewers, the authors should elaborate on potential confounding variables (e.g., diet, physical activity, season time, etc.) that could have influenced or biased the findings.

**Answer 18:** We have added a paragraph mentioning these possible confounders in the discussion section (lines 507 - 520, clean version): “While this study provides valuable preliminary insights into creatine supplementation in dialysis patients, several uncontrolled variables may have influenced the results. Dietary variations, particularly protein intake and consumption of creatine-rich foods, were not recorded during the study and could have contributed to baseline differences between participants. Also individual physical activity levels that naturally vary among individuals were not registered, representing another potential confounding factor. Additionally, seasonal variations during summer months may have influenced hydration status and vitamin D levels. With these possible confounders in mind, throughout the course of the trial, all patients received nutritional counseling provided by the same dietitian. They were also told to keep their baseline, regular physical activity levels during the study. Although these uncontrolled factors introduce some uncertainty in attributing outcomes only to creatine supplementation, they reflect real-world conditions and do not necessarily invalidate the findings, but rather highlight areas for standardization in future controlled trials”.

- Additional information that should be referenced in the discussion to support the safety of creatine supplementation on renal health includes:

<https://pubmed.ncbi.nlm.nih.gov/31375416/>
<https://pubmed.ncbi.nlm.nih.gov/38869518/>
<https://pubmed.ncbi.nlm.nih.gov/38874125/>

**Answer 19:** We thank the editor for pointing out these articles. They have been included. See references 24 -26 .

- Please provide a short paragraph of limitations before conclusions.

**Answer 20:** Limitations of this study have been incorporated before stating conclusion (489 - 506, clean version)

- Acknowledge conflicts of interest (if any) in the acknowledgements section.

**Answer 21:** The authors have declared that no competing interests exist. This statement has been added in the acknowledgements section. Line 533, clean version.

- Replace the "STROBE checklist" with "CONSORT for feasibility and pilot studies checklist"

**Answer 22:** We have replace the previous checklist with the "CONSORT for feasibility and pilot studies checklist"

==============================

Reviewer #1: Peer Review: "Oral Creatine in Hemodialysis Patients Increases Physical Functional Capacity and Muscle Mass"
Below is my peer review of the manuscript entitled "Oral Creatine in Hemodialysis Patients Increases Physical Functional Capacity and Muscle Mass." This study is very interesting and timely. However, the authors need to address some of my concerns prior to acceptance for publication.
Major Concerns:
1. Absence of a Control Group
• Description: The study employs a single-arm, before-and-after design without a control group. All 18 participants received creatine supplementation (5 g/day for 8 weeks), and outcomes were compared pre- and post-intervention without a comparator group receiving placebo or standard care.

• Reference:
• Methods section, "Patient Selection" (Page 14, Lines 124-137): "All the recruited patients received creatine supplementation. They were measured before (pre-) and after (post-) receiving creatine supplementation."
• Introduction (Page 13, Line 118): It is an observational study.
• Impact: The lack of a control group is a significant methodological flaw that compromises the study's internal validity. Without a control, it is impossible to attribute the observed improvements in skeletal muscle mass, intracellular water, phase angle, and Short Physical Performance Battery (SPPB) scores solely to creatine supplementation. Confounding factors such as the placebo effect, regression to the mean, natural recovery, seasonal variations (e.g., vitamin D levels increased from winter to summer, Page 21, Lines 241-245), or increased attention from researchers could explain the results. This limitation undermines the study's ability to establish causality, a critical aspect for a research article claiming that creatine "increases" functional capacity and muscle mass, as stated in the title and conclusion (Page 11, Lines 60-63).
• Significance: For a pilot study, a single-arm design may be exploratory, but the absence of a control group severely limits the strength of the conclusions, especially given the definitive language used (e.g., "improved muscular and functional outcomes," Page 11, Line 61).

• Recommendation: Revise the manuscript to explicitly acknowledge the lack of a control group as a significant limitation in the Discussion section (beyond the brief mention on Page 28, Line 400). Temper the conclusions to reflect the preliminary nature of the findings, e.g., change "increases physical functional capacity and muscle mass" (Title, Page 1) to "may increase" or "is associated with increases in." Suggest a randomized controlled trial (RCT) as the next step to confirm causality.
• Rationale: This adjustment aligns the interpretation with the study's observational design and mitigates overstatement, preserving credibility.

**Answer 1, Reviewer #1:** We agree with the reviewer's suggestion. The manuscript now explicitly acknowledges that this is a pilot study with a single-arm, before-and-after design, without control group (line 30, 96, 105, 332, clean version). Also, the title has been changed to reflect this suggestion and the language about our findings has been tempered.

2. Insufficient Sample Size
• Description: The study calculated a required sample size of 25 patients based on an effect size of 0.6, alpha of 0.05, and power of 0.8 for detecting changes in SPPB scores (Page 17, Lines 182-190). However, only 19 patients were recruited, and 18 completed the study, falling short of the planned sample size.
• Reference:
• Methods section, "Sample size calculation" (Page 17, Lines 182-190): "The required sample size... is 25 patients... we were only able to approach the calculated sample size."
• Results section, "Baseline Characteristics" (Page 19, Lines 210-216): "We were initially able to recruit only 19... From this initial selection, we had to withdraw one patient."
• Impact: Failing to meet the calculated sample size raises concerns about the study's statistical power. Although significant results were reported (e.g., SPPB p=0.043, skeletal muscle mass p=0.009, Page 22, Table 2), an underpowered study increases the risk of type II errors (missing true effects) and may inflate the risk of type I errors (false positives), particularly with borderline p-values like 0.043 for SPPB.
• Significance: The small sample size, combined with the lack of a control group, amplifies doubts about the robustness of the conclusions. The authors acknowledge this limitation (Page 28, Line 399), but the shortfall still constitutes a significant concern given the study's reliance on statistical significance to support its claims.
• Recommendation: Expand the Discussion to detail the implications of the reduced sample size on statistical power and result reliability (beyond the current note on Page 28, Line 399). Report post-hoc power analyses for key outcomes (e.g., SPPB, skeletal muscle mass) to clarify the strength of the findings. Recommend future studies with adequate sample sizes based on the observed effect sizes (e.g., 0.53 for SPPB, Page 25, Line 335).
• Rationale: Transparency about power limitations strengthens the manuscript's integrity and guides future research design.

**Answer 2, Reviewer #1:** We thank the reviewer for raising this point. As the editor and other reviewers suggested based on this concern, we changed our statistical analysis from non parametric tests to robust statistics. We estimated the mean differences between before and after the intervention using a bootstrap analysis with 10,000 resample. This method was used to detect statistically significant changes in variables related to both the primary and secondary outcomes, as well as in exploratory variables obtained from laboratory tests performed on the participants.

3. Selection Bias Due to Convenience Sampling
• Description: The study used a convenience sample of patients selected based on "good adherence to pharmacological and dialysis therapy" (Page 14, Lines 125-126). This non-random selection method resulted in a cohort that may not represent the broader hemodialysis population.
• Reference:
• Methods section, "Patient Selection" (Page 14, Lines 124-131): "A convenience sampling... primarily based on their good adherence to pharmacological and dialysis therapy."
• Results section, "Baseline Characteristics" (Page 19, Lines 210-216): Describes recruitment challenges, noting exclusions due to mobility issues and poor adherence.
• Impact: Selecting patients with good adherence introduces selection bias, as these individuals may be healthier, more compliant, or more motivated than the average hemodialysis patient. This bias limits the external validity of the findings, as the results may not generalize to the broader population, particularly those with poorer adherence or more severe conditions who might respond differently to creatine supplementation. The manuscript does not adequately address how this selection impacts the applicability of the findings to clinical practice.
• Significance: This methodological flaw questions the study's relevance to the target population stated in the introduction (Page 13, Lines 88-93), where hemodialysis patients are described as a vulnerable group with high morbidity and mortality. The biased sample weakens the claim that creatine could mitigate these outcomes across all hemodialysis patients (Page 11, Lines 61-63).
• Recommendation: Discuss the potential impact of selection bias in the Discussion section, noting that patients with good adherence may differ from the general hemodialysis population in health status or behavior. Adjust claims about generalizability (e.g., Page 11, Lines 61-63) to specify the study population (e.g., "in adherent hemodialysis patients"). Propose stratified sampling or broader inclusion criteria in future studies.
• Rationale: Acknowledging and contextualizing this bias enhances the manuscript's applicability and prevents overgeneralization.

Conclusion
The manuscript presents valuable preliminary data suggesting that creatine supplementation may benefit hemodialysis patients by improving muscle mass and functional capacity. However, the identified significant concerns—lack of a control group, insufficient sample size, and selection bias—fundamentally undermine the study's ability to establish causality, reliability, and generalizability. These issues do not invalidate the research entirely, especially given its pilot study context, but they require substantial revision to ensure scientific soundness.
Additional Recommendation for Future Research
Consider a double-blind RCT with a placebo control group, a more prominent and more representative sample, and monitoring of confounders (e.g., physical activity, diet) to address all three concerns comprehensively.

**Answer 3, Reviewer #1:** We thank the reviewer for his thoughtful suggestions. All of them have been incorporated in our manuscript, especially the potential bias of our sampling method (discussion, lines 493-496, clean version)

Reviewer #2: This study evaluated the effect of 8 weeks of daily creatine monohydrate supplementation in patients with end stage CRF on dialysis.
The study reported improvements in SPPB and ICW with an increase in FFM and muscle mass.
Comments to consider:

1. The study was open label and there was no control group. As the authors indicated, most subjects started in the winter and finished in the summer. This was likely one contributor to the higher vitamin D (in addition to the 8 who were supplemented) BUT was also likely associated with more physical activity. Most healthy people and patients tend to do more physical activity in the summer vs winter and this could confound the SPPB scores. The trend for those who had increased (I am assuming that the levels increased over time and not increased above normal) was rather strong (P = 0.085) suggesting that vitamin D could be a factor (keep in mind that the sample size was small for the sub-group).

**Answer 1, Reviewer #2:** We agree with the reviewer's comment and this point was specifically revised under our new statistical analysis considering robust statistics. As shown in **Supporting information: S1 Table,** bootstrap analysis in patients who increased their vitamin D levels didn’t show SPPB increases, thus suggesting that the changes observed in vitamin D were not directly related to the functional improvements observed in our group. Nevertheless we are aware that vitamin D levels have been related to muscle and functional outcomes in the literature. So, this variable will be monitored in next studies attempting to include patients that exhibit vitamin D levels in the normal range and/or controlling for the supplementation of vitamin D.

2. I do not see that the authors instructed the patients to control for diet, hydration status and pre-measurement exercise that can confound the BIA measurements.

**Answer 2, Reviewer #2:** This observation is correct. We did not instruct the patients to control for diet, hydration or exercise during the study. The decision for not doing so was based on literature findings that demonstrates that self-monitoring has significant impacts on the measured variables inducing changes on dietary intake and physical activity (Burke LE, Wang J, Sevick MA. Self-monitoring in weight loss: a systematic review of the literature. J Am Diet Assoc., 2011: 111(1):92-102. doi: 10.1016/j.jada.2010.10.008; Tang, HB., Jalil, N.I.A., Tan, CS. *et al.* Why more successful? An analysis of participants’ self-monitoring data in an online weight loss intervention. BMC Public Health, 2024: 24, 322. doi.org/10.1186/s12889-024-17848-9) (cited also in answer #6 of this rebuttal letter, editor “major details”).

Regarding control for diet, hydration or exercise before BIA measurements, all assessments were conducted in a fasting state, with participants advised to refrain from strenuous exercise for at least two hours beforehand. Notably, all measurements were obtained in the early morning hours, prior to 10:00 a.m. See lines 158-161, clean version

3. The lack of change in body weight and a ~ 2 kg increase in FFM implies that body fat was down ~ 2 kg - the authors need to comment on this and provide some explanation.

**Answer 3, Reviewer #2:**

Thank you for your comment. We have included an explanation in lines 449 - 463 clean version. In summary, the observed changes in body water compartments and muscle mass must be interpreted with the understanding that body weight measurements in this population are known to exhibit variability and are not exact (see reference 47). Additionally, water compartments—particularly extracellular water—undergo significant fluctuations during each dialysis session due to ultrafiltration, which is precisely aimed at maintaining dry weight and controlling extracellular volume. Therefore, the observed increase in fat-free mass does not necessarily imply a proportional decrease in fat mass. Rather, this increase likely reflects an expansion in intracellular water (ICW). In other words, bioelectrical impedance analysis estimates a higher fat-free mass, in part, because this tissue contains more water—specifically, more ICW.

4. The authors should discuss the limitations of the BIA and the lack of another method for the analysis such as DEXA.

**Answer 4, Reviewer #2:** Dual-energy x-ray absorptiometry (DEXA) has been suggested to be the more reliable method to measure body composition in hemodialysis population. It is able to estimate bone mineral, fat, and lean body mass directly. In comparison, bioelectrical impedance analysis (BIA) is supposed to be more affected by hydration status although according to a study conducted in patients with End-Stage Renal Disease in hemodialysis, BIA was a reliable method to perform nutritional measurements in these patients, specifically measuring total body water and “body cell mass” when compared to DEXA and other methods (Chertow et al., Nutritional assessment with bioelectrical impedance analysis in maintenance hemodialysis patients. J Am Soc Nephrol. 1995;6(1):75-81. doi: 10.1681/ASN.V6175; Fürstenberg et al. Comparison of Multifrequency Bioelectrical Impedance Analysis and Dual-Energy X-ray Absorptiometry Assessments in Outpatient Hemodialysis Patients. Am J Kidney Diseases, Volume 57, Issue 1, 123 - 129. DOI: 10.1053/j.ajkd.2010.05.022).

Besides these theoretical considerations, BIA has the advantage that it is easily performed (at the bedside or in the hemodialysis center) in comparison to DEXA (that uses x-ray irradiation and therefore requires a special infrastructure), and less expensive. Finally a pure practical issue is that this study was performed in an isolated region of Chile (Aysén Region) that lacks access to DEXA equipment.

The theoretical considerations have been incorporated in the discussion section and now it reads: “ Finally, although there are more precise methods for measuring muscle mass, such dual energy X-ray absorptiometry (DEXA) [(56)](https://www.zotero.org/google-docs/?OKxSlQ) and isotope dilution techniques for the assessment of body water compartments [(57)](https://www.zotero.org/google-docs/?78F0uu), they are unfortunately not currently accessible in our region. Therefore, we relied on bioelectrical impedance analysis (BIA), given its significantly greater accessibility and widespread clinical use worldwide. However, it would be of interest to confirm these findings using those reference techniques in settings where such technology is available. Future research involving a larger cohort and a control group is necessary to establish a definitive relationship between creatine supplementation and functional outcomes in dialysis patients” (discussion, lines 498-506, clean version).

Reviewer #3: This is a very interesting study. The hypothesis is well founded and based on relevant literature and the methods and results are sound.
In my opinion, this study should certainly be accepted for publication, provided some minor revisions are made.
The primarily limitation is the open label nature of the study, but this issue can be resolved by sufficiently stating this.

Comments:
Comment 1: Please include a short mention in the methods what an increase in phase angle represents.

**Answer 1, Reviewer #3:** This explanation has been included in the updated version: materials and methods, lines 164-166, clean version.

Comment 2: The open label nature of the study should be reflected in the abstract. Please include the words “open label” or state there was no control group.

**Answer 2, Reviewer #3:** We thank you for your suggestion. Now the words “open label” are included in the title and also expressly throughout the article.

Comment 3: The term protein-energy-wasting is not mentioned in the introduction or discussion, please incorporate this too.

**Answer 3, Reviewer #3:** We appreciate your suggestion to incorporate this important concept, which is highly relevant to the health of dialysis patients. We have included it in both the Introduction and the Discussion sections of the manuscript.

Comment 4: “Different nutritional supplements have also been explored, However, its implementation is often associated with higher costs and the need for trained personnel to ensure its proper operation.”, please elaborate which supplements are referred to with this statement, or omit altogether.

**Answer 4, Reviewer #3:** We thank the reviewer for making it evident that this point required more detail. However, based on the changes in the updated version of the article, we no longer refer in the discussion to other nutritional supplements, so this sentence has been removed.

Comment 5: The quality of the figures appears to be quite low. Please improve to 300 dpi.

**Answer 5, Reviewer #3:** We regret that the figures are not shown in the resolution we care to upload. We will take care of this important detail when sending our corrections.

Comment 6: How was intracellular water calculated? Please add.

**Answer 6, Reviewer #3:** Intracellular water (ICW) was calculated by subtracting extracellular water (ECW) from total body water (TBW). We have addressed this point in line 163. TBW and ECW are both directly obtained by BIA (Tobias A, Ballard BD, Mohiuddin SS. Physiology, Water Balance. [Updated 2022 Oct 3]. In: StatPearls [Internet]. Treasure Island (FL): StatPearls Publishing; 2025 Jan-. Available from: https://www.ncbi.nlm.nih.gov/books/NBK541059/)

Comment 7: How was missing data handled in the analyses?

**Answer 7, Reviewer #3:** We thank the reviewer for raising this question. As indicated in the results section, from this initial selection, we had to withdraw one patient because he did not attend the measurements on the scheduled dates and did not follow the creatine supplementation instructions. Since this occurred at an early stage of project development, this patient's data were not reached to be included in any of the analysis of the results and the total sample was considered as n=18.

Comment 8: Please define how dry weight was determined.

**Answer 8, Reviewer #3:** Thank you for allowing us to clarify this point. “Dry weight” is commonly defined as the post-dialysis body weight at which the patient is normotensive and free of signs or symptoms of fluid overload or depletion. In this study, dry weight was clinically estimated based primarily on physical examination, blood pressure values, and patient-reported symptoms, (lines 182-183).

Comment 9: Please report in SI units, i.e. μmol/L rather than mg/dL.

**Answer 9, Reviewer #3:** We understand the reviewers comment about this point but, after careful consideration and discussion, we decide to ask the reviewer to consider our justification for maintaining the units as they are originally reported, based on the following points:

Laboratory values in this study are reported in mg/dL rather than the international standard units (mmol/L) to align with clinical practice standards in Chile, Latin America, United States and some countries in Europe, where mg/dL remains the predominant unit used in routine clinical laboratories and medical practice. We took this decision based on an easier interpretation of results by Latin American healthcare professionals and also enhancing the clinical applicability of our findings within the regional context. The use of familiar units is particularly important for translating research findings into clinical practice, as reference ranges, diagnostic thresholds, and treatment guidelines in our setting are established in mg/dL.

Furthermore, there is extensive scientific literature in nephrology and dialysis research that employs mg/dL units, particularly in studies originating from the United States, Latin America, and other countries where these units constitute the clinical standard. This demonstrates that the use of mg/dL is not only clinically appropriate but also scientifically valid and widely accepted in dialysis literature. Major nephrology guidelines, including KDIGO 2024 guidelines report calcium, phosphorus, and other biochemical parameters in mg/dL.

Other points for justifying our decision are that maintaining consistency with previous studies conducted in similar populations and regions facilitates direct comparison of results and enables future meta-analyses. Lastly, we performed a fast search in PLOS One published papers, where mg/dL units are frequently used.

Comment 10: There is no mentioning of side effects in the results section (only shortly in the discussion). Please report on this in the results section too. Did patients experience for instance bloating/nausea or interdialytic weight gain due to the extra volume?

**Answer 10, Reviewer #3:** We thank the reviewer for raising this point. A paragraph mentioning the presence or absence of side effects has been added to the manuscript (lines 242-243, clean version).

Comment 11: Do you have any explanation why no effect was seen on handgrip strength, although this was hypothesized? Please discuss this.

**Answer 11, Reviewer #3:** Increases in handgrip strength were hypothesized based on literature obtained in different clinical settings (Alzheimer’s disease, old populations with and without chronic diseases) (Smith, A.N., Sullivan, D.K., Kelly, E., Arora, T.N., Waitsman, F.N. and Taylor, M.K. Preliminary grip strength data from an 8-week pilot trial of creatine monohydrate supplementation in Alzheimer’s disease patients. Alzheimer's Dement. 2024; 20: e092602. doi.org/10.1002/alz.092602; Davies TW, Watson N, Pilkington JJ, McClelland TJ, Azzopardi G, Pearse RM, Prowle J, Puthucheary Z. Creatine supplementation for optimization of physical function in the patient at risk of functional disability: A systematic review and meta-analysis. JPEN J Parenter Enteral Nutr. 2024; 48(4):389-405. doi: 10.1002/jpen.2607) and diverse creatine supplementation strategies. Because this measure has received much attention as a potential marker for morbidity and mortality, we decided to test if it could show improvements after our 8 week creatine supplementation intervention. We confirmed that handgrip strength did not change after creatine supplementation, after re-running our statistical analyses (see answer 10, editor). Under this finding and your suggestion, we have incorporated a paragraph in the discussion section, that reads as follows (line 393-404,clean version): Handgrip strength is a measure that deserves attention in order to its relationship to all cause mortality in hemodialysis patients (Seo-Hyeon Hwang, Dong Hoon Lee, Jihee Min, Justin Y. Jeon, Handgrip Strength as a Predictor of All-Cause Mortality in Patients With Chronic Kidney Disease Undergoing Dialysis: A Meta-Analysis of Prospective Cohort Studies, Journal of Renal Nutrition, Volume 29, Issue 6, 2019, Pages 471-479, doi.org/10.1053/j.jrn.2019.01.002). In one of the few articles referring to creatine supplementation in dialysis patients, which evaluated changes in grip strength after one year of creatine plus neuromuscular electrical stimulation, significant increases in handgrip strength were observed (Marini ACB, Pimentel GD. Creatine supplementation plus neuromuscular electrical stimulation improves lower-limb muscle strength and quality of life in hemodialysis men. Einstein (Sao Paulo). 2020 Nov 27;18:eCE5623. doi: 10.31744/einstein_journal/2020CE5623). Our findings show that after 8 weeks of creatine supplementation handgrip strength did not increase in the whole group. As mentioned earlier, the gender subgroup analysis showed significant increases in handgrip strength in men, but this observation has to be re-tested in future studies. As previous literature supporting this hypothesis came from patients in a different clinical context, the lack of a significant increase in handgrip strength in our whole group analysis could have been expected as we supplemented for a shorter period and did not add an exercise intervention as informed in Marini's work.

Comment 12: Line 86, omit additional space; Line 100, double end of sentence point after [8-9]

**Answer 12, Reviewer #3:** Thanks, we have corrected these typographical errors.
